# The MADS and the Beauty: Genes Involved in the Development of Orchid Flowers

**DOI:** 10.2174/138920211796429754

**Published:** 2011-08

**Authors:** Serena Aceto, Luciano Gaudio

**Affiliations:** Department of Biological Sciences, University of Naples Federico II, Via Mezzocannone 8, 80134 Napoli, Italy

**Keywords:** Flower development, MADS-box genes, Orchidaceae, Orchid code.

## Abstract

Since the time of Darwin, biologists have studied the origin and evolution of the Orchidaceae, one of the largest families of flowering plants. In the last two decades, the extreme diversity and specialization of floral morphology and the uncoupled rate of morphological and molecular evolution that have been observed in some orchid species have spurred interest in the study of the genes involved in flower development in this plant family. As part of the complex network of regulatory genes driving the formation of flower organs, the MADS-box represents the most studied gene family, both from functional and evolutionary perspectives. Despite the absence of a published genome for orchids, comparative genetic analyses are clarifying the functional role and the evolutionary pattern of the MADS-box genes in orchids. Various evolutionary forces act on the MADS-box genes in orchids, such as diffuse purifying selection and the relaxation of selective constraints, which sometimes reveals a heterogeneous selective pattern of the coding and non-coding regions. The emerging theory regarding the evolution of floral diversity in orchids proposes that the diversification of the orchid perianth was a consequence of duplication events and changes in the regulatory regions of the MADS-box genes, followed by sub- and neo-functionalization. This specific developmental-genetic code is termed the “orchid code.”

## THE ORCHIDACEAE: FLOWER MORPHOLOGY, PHYLOGENY AND EVOLUTIONARY ORIGIN

Among the flowering plants, the Orchidaceae family is one of the largest and includes species with greatly diversified and specialized floral morphology. Orchids have successfully spread to almost every habitat, exhibiting a broad assortment of epiphytic and terrestrial adaptations. The causes determining the wide species diversity in orchids have not been fully elucidated. However, relevant roles have been attributed to epiphytism, highly diversified pollination strategies [1], natural selection and genetic drift [2].

**Fig. (1):**
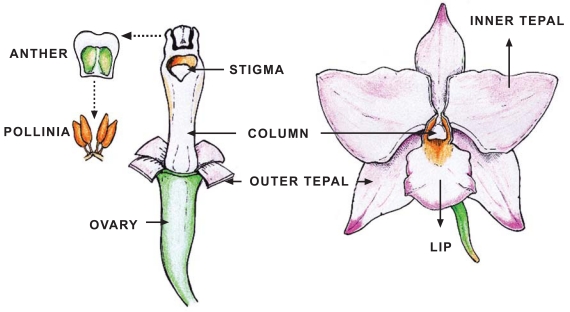
Schematic diagram of an orchid flower.

Universally known for its beauty and fascinating complexity, the orchid flower is bilaterally symmetrical (zygomorphic) and includes three outer tepals (sepals), two lateral inner tepals (petals) and a highly modified median inner tepal (lip or labellum) (Fig. **1**). The gynostemium or column is the orchid’s reproductive structure and consists of fused male (stamen/anther) and female (pistil/stigma) tissues. At the top of the column are the pollinia, packets of mature pollen grains, and at the base of the column is the ovary, which develops when triggered by pollination [3-5]. The outer tepals either can be unlobed and without ornamentations, can resemble the lateral inner tepals, or can form nectar spurs. The lip generally exhibits a distinctive shape and color pattern different from that of the other tepals and can be decorated with calli, spurs and glands. The lip is considered homologous to the adaxial tepal of other monocots and, therefore, should be the uppermost one; however, the 180° rotation in floral orientation that occurs during the development of the orchid flower (resupination) shifts the lip to the lowest tepal position. The orientation of the lip after resupination and its collocation opposite to the fertile anther suggest that its highly diversified shape and pigmentation are the result of adaptations to specific pollinators [6].

**Fig. (2):**
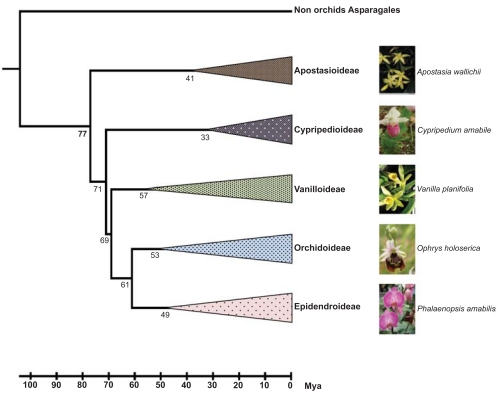
Time-calibrated phylogenetic relationships of the five sub-families of Orchidaceae, modified from Gustafsson *et al*. [14]. The numbers below the branches indicate the divergence time, as expressed in millions of years ago (Mya). On the right are the images of orchid species that are representative of each subfamily.

Before the recombinant DNA era, the phylogeny of Orchidaceae was based on a relatively small set of morphological characteristics. However, many phenotypic traits, especially the morphology of some floral structures involved in the interactions between orchids and pollinators, are of an adaptive nature and may not, therefore, reflect the family’s actual phylogeny. As a consequence, several contradictory taxonomic and phylogenetic reconstructions of the Orchidaceae have been proposed, which are reviewed in another article [7]. In recent years, data from molecular markers have been progressively added to the morphological ones, expanding and improving the body of research concerning the phylogeny of orchids. The current classification system designates five subfamilies within the Orchidaceae: Apostasioideae, Cypripedioideae, Epidendroideae, Orchidoideae and Vanilloideae (Fig. **2**). Each subfamily includes a large number of tribes and subtribes [8, 9].

In addition to the debate on the phylogeny of orchids, the last decade has witnessed intense disagreement regarding the temporal origin and diversification of this plant family. The origin of the modern orchid lineage has been placed within a wide time period ranging from ~26 to ~110 million years ago (Mya) [10-13]. The recent discovery of a fossil of *Proplebeia dominicana*, an extinct stingless bee dated 15-20 Mya, covered with pollinia from the orchid species *Meliorchis caribea* has enabled researchers to narrow the timeframe of the orchid family’s origin, estimating it at 76-84 Mya in the Late Cretaceous [12]. A more recent study [14], which includes two new orchid fossils assigned to genera *Dendrobium* and *Earina* [15], confirms the ancient origin of the orchids’ most recent common ancestor in the Late Cretaceous (~77 Mya), although the origin of the five orchid subfamilies is dated ~1-8 Mya prior to the previous estimates [14] (Fig. **2**). Both calibration analyses [12, 14] were conducted using molecular phylogenetic reconstructions based on plastid DNA sequences (*matK* and *rbcL*), highlighting the relevance of molecular analyses in the study of the origin and evolution of orchids.

Although orchids possess many traits that are “unique” in the plant kingdom, such as highly specialized pollination strategies, diversified flower morphology, peculiar ecological strategies and developmental reproductive biology, molecular studies on this family are scarce when compared with those of other species-rich plant groups [16]. The genome projects of two orchid species, *Phalaenopsis aphrodite* (Project ID 53151) and *P. equestris* (Project ID 53913), are under development, although they are not yet available for release. The OrchidBase is a freely available collection of expressed nucleotide sequences that provides integrated information on ESTs from *Phalaenopsis* orchids (http://lab.fhes.tn.edu.tw/ est) [17]. The establishment of such public resources is important, as it can facilitate the experimental design of studies on orchid biology.

In this review, we will examine the molecular mechanisms underlying the development of the flower, which is the most specialized and diversified orchid structure, with a particular emphasis on the role played by the MADS-box genes in the formation and evolution of the floral organs.

## THE GENETICS OF FLOWER DEVELOPMENT: THE MADS-BOX GENES FAMILY

The acronym MADS box is derived from the initials of four loci, *MCMI* of *Saccharomyces cerevisiae*, *AG* of *Arabidopsis thaliana*, *DEF* of *Antirrhinum majus* and *SRF* of *Homo sapiens*, all of which contain the MADS-box domain, a conserved 56-amino-acid DNA-binding domain [18]. The MADS-box family has evolved from a region of the topoisomerase II subunit A [19] and includes genes encoding transcription factors. The MADS-box genes are present in nearly all major eukaryotic groups, although they constitute a large gene family only in land plants. A gene duplication preceding the divergence of plants and animals gave rise to two main groups of MADS-box genes: type I and type II, which are distinguished on the basis of genomic organization, evolutionary rate, developmental function and level of functional redundancy [20]. The type I genes are divided into three groups, Malpha, Mbeta and Mgamma [21], and are involved predominantly in development of seed, embryo and female gametophyte [22]. The type II genes share a conserved MIKC structure and encode proteins bearing the highly conserved DNA-binding MADS domain (M) at the amino terminus, a poorly conserved I domain and a moderately conserved K domain in the central portion, which are important for protein–protein interactions and the formation of coiled-coil structures, and a variable carboxyl-terminal (C) region that may function as a transactivation domain [23, 24]. The type II genes can be further divided into MIKC^C^ and MIKC* genes, which are distinguished by their various intron/exon structures in the I domain [25]. Functional studies have suggested a major specialization of the MIKC* genes in the development of the male gametophyte [26], whereas the MIKC^C^ genes, the best-characterized group of MADS-box genes, which are often referred to simply as the MIKC genes, are involved in many functions related to plant growth and development and are closely linked to the origin of the floral organs and fruits of angiosperms. The genomic organization of the MIKC^C^ genes is generally consistent, with the presence of seven introns and eight exons [24, 27-31].

The regulatory systems controlling the expression of the MADS-box genes include complex feedback and feed-forward networks, which are often integrated in a complex cascade of events [24, 29, 32]. In addition, more specialized mechanisms, such as regulation by small RNAs [33] and epigenetic control [34], have evolved to control the expression of the MADS-box genes. In the future, more data from genome projects and reverse genetic studies will allow us to understand in greater detail the origin and functional diversification of members of this dynamic family of transcription factors [35].

**Fig. (3):**
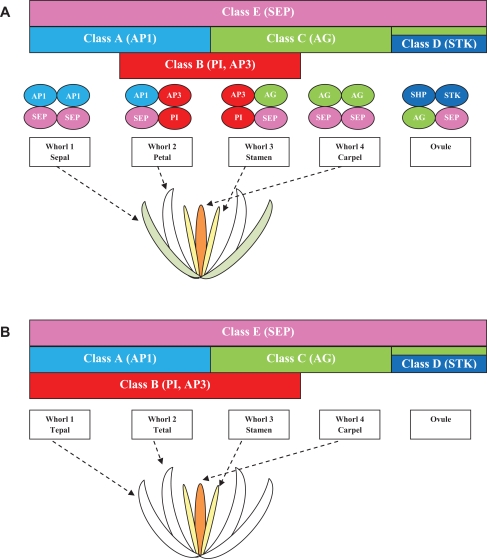
Diagram of the ABCDE and the quartet models (**A**) and of the expanded ABCDE model of floral development (**B**).

The spatial and functional activity of the floral homeotic genes is exemplified by the elegant ABCDE model of flower development (Fig. **3A**) [36, 37]. This model was initially developed on the basis of mutant analyses of the model species *Arabidopsis thaliana*, which exhibits a flower consisting of four concentric whorls of floral organs. With the exception of *APETALA2* (*AP2*), all genes involved in the ABCDE model are MADS-box genes belonging to various functional classes. In *Arabidopsis*, the expression of the class A genes (*APETALA1*, *AP1*) controls the sepal development in whorl 1 and, together with the expression of the class B genes (e.g. *PISTILLATA*, *PI*, and *APETALA3*, *AP3*) in whorl 2, regulates the formation of petals. The expression of the class B genes in whorl 3, together with the expression of the class C genes (e.g., *AGAMOUS*, *AG*), mediates stamen development. The expression of the class C genes alone in whorl 4 determines the formation of carpel. The class D genes (e.g., *SEEDSTICK*, *STK* and *SHATTERPROOF*, *SHP*) specify the identity of the ovule within the carpel, and the class E genes (e.g., *SEPALLATA*, *SEP*), expressed in the entire floral meristem, are necessary for the correct formation of all of the floral organs.

The activity of the MADS-box transcription factors requires the formation of homo- and heterodimers that recognize the conserved nucleotide CC(A/T)_6_GG DNA sequences, which are known as the CArG boxes [23]. After the formation of dimers, MADS-box proteins further interact, leading to the formation of the “floral quartets”, complexes that activate floral organ-specific expression programs [38]. For example, the quartet model predicts that the complexes AP1/AP1/SEP/SEP, AP1/SEP/AP3/PI, AG/SEP/AP3/PI and AG/AG/SEP/SEP are present within whorls 1, 2, 3 and 4, respectively, to induce the formation of floral organs (Fig. **3A**) [38, 39].

Although the ABCDE model is generally conserved [40-44], the increasing identification and functional and evolutionary analysis of the MADS-box genes is highlighting relevant differences in the mechanisms leading to flower development in non-model species, such as orchids, often emphasizing instances in which the MADS-box gene’s function could not be extrapolated from structural orthology [45]. Table **1** lists most of the MADS-box genes characterized in orchids, in Fig. (**4**) their evolutionary relationships are presented, and functions are described in the following sections.

## THE ORCHID MADS-BOX GENES OF THE *AP1/AGL9* GROUP

The *AP1*/*AGL9* group includes the phylogenetically related MADS-box genes of class A and class E [29, 38], which originated during evolution after several duplication events [46, 47]. Class A genes belong to the *AP1*/*SQUA*-like subfamily (from the *APETALA1* and *SQUAMOSA* locus of *Arabidopsis thaliana* and *Antirrhinum majus*, respectively), which is further divided into the paleo*AP1*-like and the eu*AP1*-like clades [48, 49]. The C-terminal region of the AP1/SQUA-like proteins exhibits conserved motifs. The paleoAP1 motif L/MPPWML (also known as the FUL-like motif, from the *FRUITFULL* locus of *A. thaliana*) is typical of the paleo*AP1*-like clade, whereas the eu*AP1*-like clade is characterized by two alternative motifs, RRNaLaLT/NLa (the euAP1 motif) and CFAT/A (the farnesylation motif), the latter evolved from the paleoAP1 motif through a frameshift mutation [48]. The role of the paleoAP1 and farnesylation motifs is not clear, and the absence of both motifs in some *AP1*/*SQUA*-like genes does not affect their function [50, 51]. As the *AP1*/*SQUA-*like genes are present only in angiosperms, their origin might be related to the emergence of the floral perianth. The eu*AP1-*like clade is typical of the higher eudicots, while the paleo*AP1 *clade is present in both monocots and dicots [48, 49].

The E-function genes belong to the *SEP*-like subfamily (from the *SEPALLATA* locus of *A. thaliana*), which are divided into *SEP3* and *SEP1/2/*4 clades (previously known as *AGL9* and *AGL2/3/4* clades, respectively) [29, 46, 47, 52]. A third clade, *AGL6*, also belongs to the *AP1*/*AGL9* group [53-56]. In addition to their role in determining floral organs, almost all of the members of the *AP1*/*AGL9* group of MADS-box genes are also involved in the floral meristem’s initiation and development [57]. This finding suggests that the genes of the *AP1*/*AGL9* group could function at the top of the regulatory hierarchy of the MADS-box genes involved in flower development [58-60]. 

**Fig. (4):**
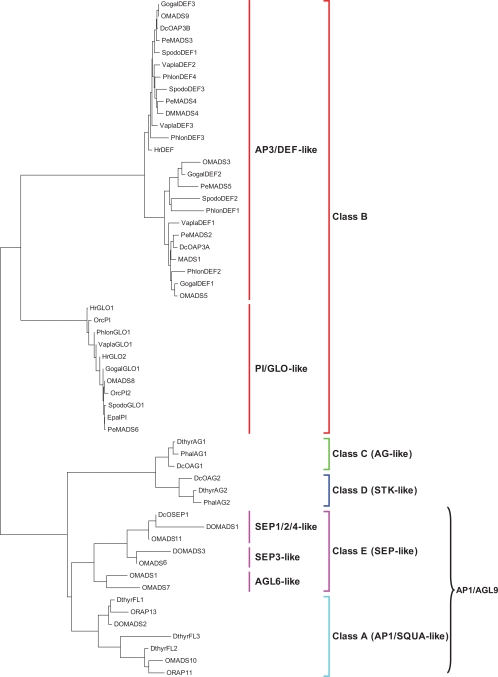
Neighbor-joining tree, obtained from the alignment of the amino-acid sequences of the orchid MADS-box proteins, outlining the various functional classes.

In orchids, a number of genes belonging to the *AP1/AGL9* group have been identified and functionally char acterized (Table **1**, Fig. **4**). The identification of genes that function early during floral transition is the first step toward the elucidation of the molecular mechanisms of floral transition in orchids.

In the orchid *Dendrobium *Madame Thong-In, the MADS-box genes *DOMADS1*, *DOMADS2 *and *DOMADS3* are homologous to *SEP1*, *AP1*/*SQUA* and *SEP3*, respectively. These genes are successively activated during the floral transition and continue to be expressed later in mature flowers [58]. Their expression pattern is quite different when compared with the transcriptional profile of the homologous genes of *Arabidopsis*, revealing an absence of functional conservation in MADS-box genes functioning during floral transition in flowering plants. *DOMADS1*, *DOMADS2* and *DOMADS3*, in accordance with almost all of the MADS-box genes involved in the regulation of floral transition, also function in the later stages of flower development [61-64]. *DOMADS1 *transcripts are present in the inflorescence meristem, in the floral primordium and in all of the floral organs. The same expression pattern in floral organs is shared by the *DOMADS1* ortholog *DcOSEP1* of *Dendrobium crumenatum* [65]. *DOMADS2 *is expressed early in the apical meristem of the shoot and throughout the process of floral transition; later, its expression is restricted to the column. The transcription of *DOMADS3 *is detectable before the differentiation of the flower primordium, and its expression in floral organs is only detectable in the pedicel tissue.

Compared with the activities in the floral transition of the *Arabidopsis* orthologs *AP1*, *AGL8* and *CAULIFLOWER* (*CAL*) [61, 66, 67], followed by the activation of *SEP1*, *SEP4* and *SEP3 *in stage 2 of the flower primordium [68-70], *DOMADS2 *and *DOMADS3 *are activated much earlier. Differences are also observed in the spatial expression pattern, as the transcripts of *DOMADS1 *and *DOMADS2 *accumulate in both the inflorescence and the floral meristem, whereas in *Arabidopsis*, the expression of *AGL8 *is restricted to the former region and that of *AP1 *and *CAL *is confined to the latter region. These differences indicate the evolution of specific regulatory systems controlling the activity of MADS-box genes involved in floral transition in various plant families.

The promoter region of the *DOMADS1 *gene contains multiple *cis*-acting elements that regulate the expression of *DOMADS1* in the orchid’s reproductive organs and, at low levels, in the stem [71]. This promoter contains six CArG-box sequences, which are the binding sites of diverse MADS-box genes and are crucial modulators of their expression [72-75]. The presence of the CArG-boxes within the *DOMADS1* promoter, as well as in the promoters of MADS-box genes of distantly related species (e.g., *Arabidopsis*), implies that the basic mechanism of regulation of the MADS-box genes through binding to the CArG-box sequences are conserved during the flowering process. In addition, within the promoter of the *DOMADS1* gene, there are five DNA-binding sites of the class 1 *knox *gene *DOH1* [71], which is a negative regulator of the expression of *DOMADS1* during floral transition that may directly interact with its binding sites to mediate the regulation of *DOMADS1 *expression [76].

In addition to *DOMADS1-3*, three other MADS-box genes belonging to the *AP1*/*AGL9* group have been characterized in *Dendrobium*. The *DthyrFL1-3* genes of *D. thyrsiflorum* are paleo*AP1*-like genes within the *AP1/SQUA-*like subfamily, which evolved from a single ancestor common to all monocots [51]. Similarly to the events driving the evolution of several MADS-box gene lineages [48, 77-79],  a frameshift mutation is considered responsible for the absence within the *DthyrFL3* locus of the paleoAP1-like motif present in both *DthyrFL1* and *DthyrFL2*. All three of the genes are transcribed at low levels in vegetative root and leaf tissues, at higher levels in ovules and at much higher levels in inflorescences, with increasing transcription levels of *DthyrFL1* and *DthyrFL2* observed from small to large floral buds [51]. This expression pattern may indicate that the genes are involved in different mechanisms controlling the development of orchid inflorescence.

In the orchid *Phalaenopsis amabilis*, the genes *ORAP11 *and *ORAP13 *belong to the *AP1*/*SQUA*-like subfamily, exhibit the typical paleo*AP1*-like motif and lack the farnesylation motif [80]. Both genes possess a role in the establishment of meristem identity, with initial expression in the inflorescence and floral meristems that is similar to the early functions of *FUL *in *A. thaliana *[81] and *OsMADS18 *in rice [82]. Later expression of both *ORAP11 *and *ORAP13 *in the primordia of all floral organs is consistent with the transcriptional profile of the genes *OsMADS18 *[82] and *LtMADS1* of the monocots *Oryza sativa* and *Lolium temulentum *[83], respectively, but not with that of the *AP1 *and *FUL *genes of *Arabidopsis* [66, 84]. Subsequently, both *ORAP *genes participate to the development of petals, lips, columns and ovules, with the last role also described for *DthyrFL1*-*3 *in *D. thyrsiflorum *[51] and *PFG *in petunia [85]. The presence of *ORAP11 *transcripts in the columns of mature flowers is consistent with the expression pattern of the *DOMADS2 *gene in *Dendrobium *Madame Thong-In [58]. *ORAP* genes are also expressed in vegetative tissues, such as the root and procambial strand region, thereby resembling the *FUL*, *PFG*, *OsMADS18 *and *LtMADS1 *genes more than the *AP1*, *SQUA *or *PEAM4*, the *AP1* homolog of pea [83-89]. The expression profile of the *ORAP *genes suggests that the orchid *AP1/SQUA*-like genes have retained an ancestral role in the determination of meristem identity, but they have functions that are quite different from those of the “classic” class A genes.

In the orchid *Oncidium* Gower Ramsey, the *OMADS1* gene belongs to the *AP1*/*AGL9* group; in particular, it belongs to the *AGL6* clade [90]. *OMADS1* is transcribed early in the apical meristem of the orchid, and its role in regulating floral initiation is functionally similar to that of other members of the *AP1*/*AGL9* group, such as *AP1* and *SEP3* [50, 91-93].  The OMADS1 protein is able to form heterodimers with OMADS3, a class B orchid MADS-box protein also involved in the process of floral initiation [94]. However, the expression pattern of *OMADS1* in the mature flower, which is restricted to the lip and carpel, does not overlap with that of its orthologs *AGL6* of *Arabidopsis* and *ZAG3* of *Zea mays*, which are expressed in all four flower organs and ovules [53, 54]. The heterodimerization activity of OMADS1 is also achieved with OMADS2, an *Oncidium* class D MADS-box protein that is expressed in the stigmatic cavity and ovary [95]. *OMADS1* may represent a class of MADS-box genes with a function similar to that of the carpel-specific MADS-box genes in regulating floral initiation and ovary development in orchids.

In *Oncidium*, four additional *AP1/AGL9*-like genes, *OMADS6*, *OMADS7*, *OMADS10 *and *OMADS11*, have been characterized [96]. Specifically, *OMADS6 *is a *SEP3 *ortholog, *OMADS11 *is closely related to the *SEP1/2 *orthologs and *OMADS7 *is closely related to *AGL6-*like genes within the E-function genes; furthermore, *OMADS10 *is a paleo*AP1 *ortholog of orchid. *OMADS6*, *OMADS7* and *OMADS11 *exhibit a similar expression pattern, whereas *OMADS10 *has a completely different profile. In contrast with the expression profile of the *SEP3* gene and many of its orthologs, which are transcribed only in the three inner whorls of the flower [27, 34, 47, 69, 97-100],  the expression of *OMADS6* is observed in all four floral whorls, exhibiting relatively low levels in stamens. This pattern is similar to that of the *SEP1/2* genes [27, 70, 100] and of *LMADS3*, a *Lilium* *SEP3* ortholog [101], and could be explained by the significant morphological similarities between sepals and petals (tepals) in orchids and lilies. The expression of *OMADS11*, which is absent in the stamens, resembles that of *OMADS6*. The expression pattern of *OMADS7 *overlaps with that of *OMADS6 *and is similar to that of *AGL6 *of *A. thaliana* and *ZAG3 *of maize [54]. However, the expression profile of *OMADS7 *is divergent from that of *OMADS1*, which exhibits an expression pattern restricted to the lip and carpel [90]. Although not identical, the similar expression patterns of the *OMADS6*, OMADS7 and OMADS*11 *genes suggest a possible evolutionary conservation of their transcriptional regulation.

Even though most genes of the AP1/*SQUA-*like subfamily are generally expressed in the early floral meristem and in floral organs and are absent in vegetative tissues [58, 66, 67, 86, 102, 103], some *AP1*/*SQUA*-like genes in monocots are also expressed in leaves [82, 104, 105]. *OMADS10 *is only expressed in the leaves, lips and carpels, and this expression pattern indicates a possible functional conservation for specific lineages of *AP1*/*SQUA*-like genes in monocots.

## THE ORCHID CLASS C AND D MADS-BOX GENES

Within the ABCDE model of flower development, the class C genes regulate the development of carpels and, together with the class B genes, of stamens. The class D genes are primarily involved in the development of ovules. The class C and D genes are sister clades, which appeared after an early duplication event during angiosperm evolution [78]. Two motifs at the C-terminus, the AG motifs I and II, are common to all of the class C and D gene products [78].

In orchids, the number of characterized genes belonging to the C and D classes is smaller than those of the other classes (Table **1**, Fig. **4**).

In *Dendrobium crumenatum*, the *DcOAG1* and *DcOAG2* genes belong to the class C and class D MADS-box genes, respectively [65]. *DcOAG1* is an ortholog of *AG* of *A. thaliana*, presents an N-terminal extension preceding the MADS domain, which is typical of the class C genes, and its genomic sequence contains the intron 8, which is common in several *AG*-like genes of class C and was possibly lost in the class D lineage after the divergence of Nympheales from the other angiosperms [78, 106]. *DcOAG2* is a *SEEDSTICK* (*STK*) homolog and is specifically expressed in the ovary. The expression of *DcOAG1* is detectable in all of the floral organs and, in accordance with the expression of the *AG* orthologs observed in some basal angiosperms, is not confined to the reproductive organs [43]. This common expression pattern shared between the *AG* orthologs of orchids and basal angiosperms indicates that the regulatory mechanisms involved in the expression of these class C genes may have evolved independently.

In *Dendrobium thyrsiflorum*, *DthyrAG1* is a class C gene and *DthyrAG2* belongs to class D [107]. Both genes encode the conserved AG motifs at the C-terminus of the protein, and *DthyrAG2* encodes an extension of the AG motif, the MD motif YET/AKA/DDXX, which is typical of the monocot D lineage genes and may be involved in determining their interaction with specific protein partners [78]. The *DthyrAG1* gene presents an intron 8 located before the stop codon. Both *DthyrAG1* and *DthyrAG2* are expressed during ovule and flower development, specifically in the rostellum, stigma and stylar canal. In monocots, class D orthologs are generally expressed in ovules [108, 109], and the dicots exhibit a similar expression pattern; however, some exceptions have been reported, such as the *LMADS2* gene of *Lilium longiflorum*, which is expressed in the stylum [110], and the *ZmZAG2* of *Zea mays*, which is expressed in the stigma [111]. The differences in expression patterns between the class D lineage genes in monocots and dicots, together with the presence in monocots of the extension of the AG motif, could be related to the acquisition of a novel function for class D genes within monocots. Both the *DthyrAG1* and *DthyrAG2* genes are also expressed during ovule development, in agreement with the genes of class C and D in other species [27, 103, 108, 112, 113]. However, *DthyrAG1* is only transcribed early, whereas *DthyrAG2* is expressed throughout the process of ovule development, suggesting a prominent role for *DthyrAG2* in late ovule development [107].

In *Phalaenopsis*, the products encoded by the genes *PhalAG1* and *PhalAG2* contain the AG I and II motifs in their C-terminal regions, and PhalAG2 also exhibits the MD motif [114]. *PhalAG1* and *PhalAG2* belong to the class C and D MADS-box genes, respectively. Both are genes are expressed in all floral organs at the earliest stage of floral development and, later, in the lip and column. Although these genes belong to different classes of AG-like genes, their similar expression patterns strongly suggest a subfunctionalization of the two genes. In contrast to the AG-like genes of the other monocots, which are generally involved in stamen and carpel development and are not expressed in whorls 1 and 2, *PhalAG1* and *PhalAG2* are also involved in the lip formation [114].

In *Oncidium *Gower Ramsey, the genes *OMADS4* and *OMADS2* belong to classes C and D, respectively [95]. Both of their encoded proteins present the AG motifs I and II, and OMADS2 also contains the conserved MD motif, which is specific to class D proteins of monocots. *OMADS4 *is specifically expressed only in stamens and carpels, thus resembling the expression patterns of other class C genes [111, 115, 116]. *OMADS2 *is only expressed in carpels, in accordance with other class D genes [117, 118]. Despite the sequence similarity, the expression patterns of *OMADS4* and *OMADS2* are quite divergent when compared with those of *PhalAG1 *and *PhalAG2* and may reflect a functional evolutionary divergence of the class C/D genes in *Oncidium* and *Phalaenopsis*, with a more redundant role in the latter species than in *Oncidium* [95].

Although only one class C gene has been identified in *Dendrobium thyrsiflorumm *(*DthyrAG1*), *D. crumenatum *(*DcOAG1*), *Oncidium* (*OMADS4*) and *Phalaenopsis *(*PhalAG1*), a duplication event generated two class C MADS-box genes in the orchid *Cymbidium ensifolium* (*CeMADS1 *and *CeMADS2*), both of which are involved in regulating the development of the gynostemium [87]. Despite their redundant function in the meristem tissue, these two paralogs exhibit temporal and spatial differences in their expression pattern in floral organs, leading to the hypothesis of sub- and neo-functionalization during the evolution of the *CeMADS1* and *CeMADS2* genes [87]. According to the floral quartet model, the function of *CeMADS1* and *CeMADS2* genes in column development is enabled through the formation of the tetrameric protein complexes CeMADS1-CeMADS1-class E-class E and/or CeMADS1-CeMADS2-class E-class E. *CeMADS1* has a pivotal role in stamen and carpel development; in fact, the *Cymbidium* naturally occurring mutant *multitepal*, in which the column is substituted by tepals, continues to express *CeMADS2* but not *CeMADS1*. The transcription of *CeMADS1 *enhances the formation of the column, followed by the expression of *CeMADS2 *to complete development correctly. The function of *CeMADS2 *is primarily maintenance, rather than initiation, and its expression alone is not sufficient to mediate the formation of the column [87].

## THE ORCHID CLASS B MADS-BOX GENES

Based on the ABCDE model, the class B MADS-box genes are necessary for the correct development of petals and stamens and include two major lineages, the *AP3/DEF*-like genes (from the *APETALA3* and *DEFICIENS* loci of *A. thaliana* and *A. majus*, respectively) and the *PI/GLO*-like genes (from the *PISTILLATA *and* GLOBOSA *loci of* A. thaliana *and* A. majus*, respectively), which appeared after a duplication of an ancestral gene containing a paleoAP3 motif [77, 119]. The *AP3/DEF*-like genes include the paleoAP3 clade and two further clades, TM6 and euAP3, which originated after a second duplication event [77].

The class B genes characterized in orchids are the most numerous and thoroughly studied compared with those of the other classes (Table **1**, Fig. **4**). A feature common to a high number of the class B MADS-box orchid genes is the expansion of their expression profile into the first whorl of floral organs that may be responsible for the development of petaloid sepals in orchids (Fig. **3B**).

In *Phalaenopsis* *equestris*, the four class B genes, *PeMADS2-5*, are AP3/*DEF*-like paralogs that are expressed during developmental stages ranging from early to late inflorescence [120]. Their organ-specific expression pattern demonstrates an absence of functional redundancy. In fact, *PeMADS2* is strongly expressed in the outer and inner tepals and, at lower levels, in the column; *PeMADS3* is strongly expressed in the inner tepals and lips and, to a lesser extent in the column; *PeMADS4* is expressed only in the lips and the column; *PeMADS5* is expressed in the outer and inner tepals, lips and the column. The expression pattern of these AP3/*DEF*-like genes in the naturally occurring *Phalaenopsis* peloric mutant reveals that *PeMADS2*, *PeMADS4 *and *PeMADS5 *are involved in specifying the development of the outer tepals, lip and inner tepals, respectively. In addition, *PeMADS4 *is also involved in column development, and *PeMADS5 *is important for the initiation of stamens [120].

In contrast to its complement of four AP3/*DEF*-like genes, the genome of *P. equestris* contains only one *PI*/*GLO*-like gene, *PeMADS6* [121]. The expression of *PeMADS6* in the inflorescence meristem and floral primordium highlights its role in initiating floral development. The expression pattern of *PeMADS6* in the outer and inner tepals, lip, column and ovary demonstrates its involvement in the development of these floral organs. Furthermore, the persistence of *PeMADS6* transcripts in the flower until senescence might correlate the activity of this gene to the flower longevity of orchids [121]. The PeMADS2-5 proteins can interact with PeMADS6 to mediate the development of specific organs [122]. In addition, PeMADS4 and PeMADS6 can form homodimers, and both the PeMADS4 homodimer and the PeMADS6 homodimer/homomultimer can bind the CArG boxes, which are the MADS-box protein-binding motif. Also, the heterodimers PeMADS2–PeMADS6, PeMADS4–PeMADS6 and PeMADS5–PeMADS6 are able to bind the CArG boxes, indicating that, in orchids, the *AP3/DEF*-like and *PI/GLO*-like proteins interact in different combinations and revealing the notable complexity of their regulatory functions [122].

In *Dendrobium crumenatum*, *DcOPI* is a class B gene belonging to the *PI/GLO*-like lineage, whereas *DcOAP3A* and *DcOAP3B* belong to the paleo*AP3* lineage of the *AP3/DEF*-like genes [65]. Both *DcOPI* and *DcOAP3A* are expressed in all whorls of the floral organs. *DcOAP3B* is expressed in inner tepals and lip, in pollinia and in the column. These three genes are also expressed in the ovary [65].

In *Habenaria radiata*, three class B MADS-box genes have been identified: *HrGLO1* and *HrGLO2*, which are two *PI/GLO*-like genes, and *HrDEF*, which is an *AP3/DEF*-like gene [123]. *HrGLO1* and *HrGLO2* are expressed in the outer and inner tepals and in the column, whereas *HrDEF* is expressed only in the inner tepals and the column [123].

In *Oncidium* Gower Ramsey, *OMADS3*, *OMADS5* and *OMADS9* are class B MADS-box genes belonging to the *AP3/DEF-*like lineage, whereas* OMADS8* is a *PI/GLO*-like gene [94, 124]. *OMADS8* is expressed in all of the floral organs and leaves. *OMADS3* is expressed in all four flower organs and in leaves, exhibiting an expression pattern similar to that of a number of the *AP3/DEF*-like genes of the TM6 clade [125]. *OMADS5* is only expressed in the outer and inner tepals, in accordance with *PeMADS2* of *Phalaenopsis* [120]. *OMADS9* is transcribed in the inner tepals and lip, in agreement with the expression patterns of *DcOAP3B*, *PeMADS3* and *HrDEF* [65, 120, 123]. Both *OMADS5* and *OMADS9* are not expressed in stamens and leaves, and their expression profiles are different from that of *OMADS3*, which is expressed in all flower organs and leaves [94], indicating a functional diversification of *OMADS5*, *OMADS9* and *OMADS3* [124]. OMADS5 can form homodimers and heterodimers with OMADS3 and OMADS9, whereas OMADS8 forms heterodimers only with OMADS3, while OMADS3 can form homodimers and heterodimers with OMADS8. OMADS9 can form homodimers and heterodimers with OMADS3 and OMADS5 [124].

In *Orchis italica*, *OrcPI* is a class B *PI/GLO*-like gene [126]. *OrcPI* transcripts are detectable in all floral organs, and the maintenance of *OrcPI* transcripts in the flower through anthesis to senescence confirms the relationship between the *PI/GLO*-like genes and the long-persisting flower longevity of orchids, as also described in *Phalaenopsis* [127]. The high number of MADS-box gene sequences publicly available has enabled comparative evolutionary studies to determine the selective constraints acting on coding and/or non-coding regions and the eventual traces of adaptive, purifying and neutral selection. Different evolutionary constraints act on the coding and non-coding regions of *OrcPI*, suggesting a heterogeneous selective pattern of the *OrcPI* locus [126, 127]. Phylogenetic footprinting analysis detected conserved regions within the 5’ regulatory sequence of *OrcPI* and the homologous regions of *Oryza sativa*, *Lilium regale* and *Arabidopsis* *thaliana*, confirming the wide conservation of regulatory signals required during flower development [128]. A paralog copy of *OrcPI*, *OrcPI2*, has been recently identified in *O. italica* and other members of the Orchidoideae subfamily. The two *PI/GLO*-like genes exhibit different selective pressures, particularly on the synonymous sites, and seem to have experienced subfunctionalization [129]. In *O. italica*, four *AP3/DEF*-like genes are also present (Aceto *et al*., unpublished data).

Recently, the evolutionary analysis of a number of class B genes from the major subfamilies of Orchidaceae indicated the presence of four distinct clades (from 1 to 4) of *AP3/DEF*-like orthologs, while the *PI/GLO*-like genes seem to form a single ancient clade with recent paralogs present only in the Orchidoideae subfamily [123, 129, 130]. Within the four *AP3/DEF*-like clades, the genes belonging to clade 2 exhibit relaxation of purifying selection when compared with the other orchid *AP3/DEF*-like clades and with the *PI/GLO*-like genes. In Orchidaceae, gene duplication followed by sub- and neo-functionalization, particularly within the class B *AP3/DEF*-like genes, seems to have played a crucial role in the morphological evolution that resulted in the extreme specialization of the floral perianth [130].

## THE ORCHID CODE

In contrast to *Arabidopsis* and the other eudicots, which exhibit sepals (whorl 1) and petals (whorl 2) with clearly different morphologies, the flowers of orchids and a number of other monocots have phenotypically similar organs (tepals) in the outer whorls 1 and 2. The modification of the ABCDE model attributes this difference to the extension of the expression of the class B genes into the whorl 1, in addition to the expression in whorls 2 and 3 [131, 132] (Fig. **3B**). However, orchid tepals are distinguished in the outer and inner tepals and, among the latter, the lip has a highly diversified morphology, which is a feature that cannot be satisfactorily explained by the modified ABCDE model.

A decade of molecular studies on the orchid MADS-box genes has strongly enhanced understanding of the mechanisms underlying flower development in this plant family. However, questions remain regarding the evolution and diversification of flower morphology in orchids. Can all of the data obtained from various orchid species be integrated into an evolutionary model to explain the uniqueness of the orchid flower? The recent theory known as “the orchid code” proposes an elegant model describing the development and evolution of the orchid perianth [6, 133, 134].

The orchid code theory illustrates a developmental-genetic code that attributes to the class B *AP3/DEF*-like genes a pivotal role in tepal and lip identity and leaves unchanged the function of the class B *PI/GLO*-like genes and the functions of the A, C, D and E class genes with respect to the modified ABCDE model.

In contrast to eudicot model species, such as *Arabidopsis*, in which the identity of petals is realized through the interaction of one *AP3/DEF*-like and one *PI/GLO*-like gene product, the orchid code theory suggests that the identity of orchid tepals and lips is determined by the interactions of the products of four paralogous *AP3/DEF*-like genes belonging to four different clades with the product of one *PI/GLO*-like gene. The orchid *AP3/DEF*-like genes are grouped into four well-defined clades: clade 1 (*PeMADS2*-like) is sister to clade 2 (*OMADS3*-like), while clade 3 (*PeMADS3*-like) is sister to clade 4 (*PeMADS4*-like). Each clade is characterized by a specific expression pattern [133, 134].

**Fig. (5):**
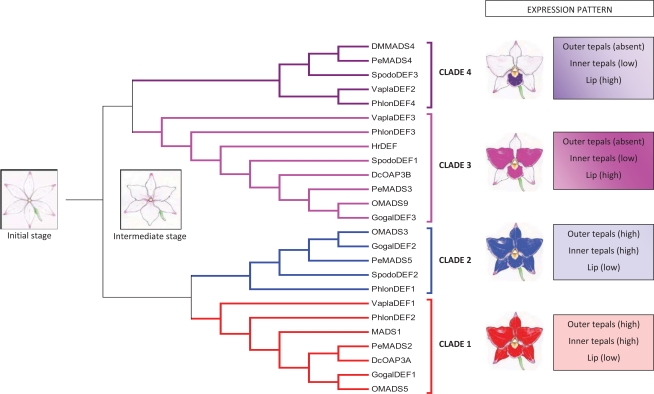
The orchid code, modified from Mondragon-Palomino and Theissen [133, 134]. The colors indicate the various clades of the *AP3/DEF*-like genes and their expression profiles in the orchid perianth. A model for the possible initial and intermediate stage of the orchid perianth is also presented. (For interpretation of the references to color in this figure legend, the reader is referred to the web version of this paper).

Under the assumptions of the orchid code theory, the interactions of the clade 1 and clade 2 gene products mediates the development of the outer tepals (whorl 1). The formation of the two lateral inner tepals (whorl 2) is specified by the interaction of high levels of the clade 1 and 2 and low levels of the clade 3 and 4 gene products, whereas the development of the lip, which is a highly modified inner tepal, is determined by the expression of high levels of the clade 3 and 4 gene products, in addition to low levels those of clades 1 and 3 (Fig. **5**). Thus, the expression of clade 3 genes differentiates between the inner and outer tepals, whereas the expression of clade 4 genes distinguishes between the two lateral inner tepals and the lip [6, 133].

This proposed scheme can also explain the evolution of the zygomorphic orchid flower, starting from an actinomorphic flower composed of six nearly identical tepals in which the ancestor of the current *AP3/DEF*-like genes was equally transcribed. The duplication and evolution of different *cis*-regulatory elements played a fundamental role in the functional diversification of the four *AP3/DEF*-like orchid clades. An initial duplication event produced the ancestor of the clade 1 and clade 2 genes and the ancestor of the clade 3 and clade 4 genes. At this stage, the evolution of a more specialized expression of the ancestor of the clade 3 and 4 genes, which was excluded from the outer tepals, might have established an intermediate flower structure, with distinctive outer and inner tepals (Fig. **5**). After a second duplication round, clade 3 and clade 4 genes differentiated, and the modularization of their expression led to the evolution of the lip [133, 134].

## CONCLUSIONS

The ancient scientific interest in the “mystery” of the orchid flower has greatly expanded since the advent of the EVO/DEVO molecular approach. Certainly, the future of studies on the flower development genes in orchids will greatly benefit from the completion of the genome projects currently in progress and from new and challenging transcriptomic projects, such as the analysis of microRNAs. The continuous and increasing characterization of genes involved in flower development in orchids has clarified many functional and evolutionary aspects of orchid development. The complexity of the expression pattern of the class B MADS-box genes of the Orchidaceae has successfully been simplified and integrated in the specific developmental-genetic code of the orchid perianth, even if the evolutionary role of the recently discovered paralogs of the *PI/GLO*-like genes still remain to be clarified. A more extensive and detailed analysis of the orchid MADS-box genes belonging to classes A, C, D and E will allow the proposal of a more exhaustive model that could also explain the evolution and diversification of the orchid’s reproductive structures. In this context and in the presence of a growing number of characterized loci, it is particularly important to establish a gene nomenclature system that is less ambiguous than the existing one to identify clearly homolog and paralog genes.

## Figures and Tables

**Table 1:** MADS-Box Genes Identified in Orchids

Locus	Species	Clade	Class	Genbank Accession Number
*DthyrFL1*	*Dendrobium thyrsiflorum*	AP1/SQUA -like	A	AY927236
*DthyrFL2*	*Dendrobium thyrsiflorum*	AP1/SQUA -like	A	AY927237
*DthyrFL3*	*Dendrobium thyrsiflorum*	AP1/SQUA -like	A	AY927238
*DOMADS2*	*Dendrobium* Madame Thong-In	AP1/SQUA-like	A	AF198175
OMADS10	*Oncidium* Gower Ramsey	AP1/SQUA-like	A	HM140846
*ORAP11*	*Phalaenopsisamabilis*	AP1/SQUA-like	A	DQ104328
*ORAP13*	*Phalaenopsisamabilis*	AP1/SQUA-like	A	DQ104327
*MADS1*	*Cymbidium *hybrid cultivar*.*	AP3/DEF-like	B	DQ683575
*DcOAP3A*	*Dendrobium crumenatum*	AP3/DEF-like	B	DQ119838
*DcOAP3B*	*Dendrobium crumenatum*	AP3/DEF-like	B	DQ119839
*DMMADS4*	*Dendrobium moniliforme*	AP3/DEF-like	B	GU132995
*GogalDEF1*	*Gongora galeata*	AP3/DEF-like	B	FJ804097
*GogalDEF2*	*Gongora galeata*	AP3/DEF-like	B	FJ804098
*GogalDEF3*	*Gongora galeata*	AP3/DEF-like	B	FJ804099
*HrDEF*	*Habenaria radiata*	AP3/DEF-like	B	AB232663
OMADS3	*Oncidium* Gower Ramsey	AP3/DEF-like	B	AY196350
*OMADS5*	*Oncidium* Gower Ramsey	AP3/DEF-like	B	HM140840
*OMADS9*	*Oncidium* Gower Ramsey	AP3/DEF-like	B	HM140841
*PeMADS2*	*Phalaenopsis equestris*	AP3/DEF-like	B	AY378149
*PeMADS3*	*Phalaenopsis equestris*	AP3/DEF-like	B	AY378150
*PeMADS4*	*Phalaenopsis equestris*	AP3/DEF-like	B	AY378147
*PeMADS5*	*Phalaenopsis equestris*	AP3/DEF-like	B	AY378148
*PhlonDEF2*	*Phragmipedium longiflorum*	AP3/DEF-like	B	FJ804106
*PhlonDEF1*	*Phragmipedium longiflorum*	AP3/DEF-like	B	FJ804105
*PhlonDEF3*	*Phragmipedium longiflorum*	AP3/DEF-like	B	FJ804107
*PhlonDEF4*	*Phragmipedium longiflorum*	AP3/DEF-like	B	FJ804108
*SpodoDEF2*	*Spiranthes odorata*	AP3/DEF-like	B	FJ804111
*SpodoDEF1*	*Spiranthes odorata*	AP3/DEF-like	B	FJ804110
*SpodoDEF3*	*Spiranthes odorata*	AP3/DEF-like	B	FJ804112
*VaplaDEF1*	*Vanilla planifolia*	AP3/DEF-like	B	FJ804115
*VaplaDEF3*	*Vanilla planifolia*	AP3/DEF-like	B	FJ804117
*VaplaDEF2*	*Vanilla planifolia*	AP3/DEF-like	B	FJ804116
*EpalPI*	*Epipactis palustris*	PI/GLO-like	B	DQ005588
*GogalGLO1*	*Gongora galeata*	PI/GLO-like	B	FJ804100
*HrGLO1*	*Habenaria radiata*	PI/GLO-like	B	AB232665
*HrGLO2*	*Habenaria radiata*	PI/GLO-like	B	AB232664
*OMADS8*	*Oncidium* Gower Ramsey	PI/GLO-like	B	HM140842
*OrcPI*	*Orchis italica*	PI/GLO-like	B	AB094985
*OrcPI2*	*Orchis italica*	PI/GLO-like	B	AB537504
*PeMADS6*	*Phalaenopsis equestris*	PI/GLO-like	B	AY678299
*PhlonGLO1*	*Phragmipedium longiflorum*	PI/GLO-like	B	FJ804109
*SpodoGLO1*	*Spiranthes odorata*	PI/GLO-like	B	FJ804114
*VaplaGLO1*	*Vanilla planifolia*	PI/GLO-like	B	FJ804118
*CeMADS1*	*Cymbidium ensifolium*	AG-like	C	GU123626
*CeMADS2*	*Cymbidium ensifolium*	AG-like	C	GU123627
*DcOAG1*	*Dendrobium crumenatum*	AG-like	C	DQ119840
*DthyrAG1*	*Dendrobium thyrsiflorum*	AG-like	C	DQ017702
*PhalAG1*	*Phalaenopsis sp.*	AG-like	C	AB232952
*DcOAG2*	*Dendrobium crumenatum*	STK-like	D	DQ119841
*DthyrAG2*	*Dendrobium thyrsiflorum*	STK-like	D	DQ017703
*PhalAG2*	*Phalaenopsis sp.*	STK-like	D	AB232953
*OMADS1*	*Oncidium* Gower Ramsey	AGL6-like	E	HM140843
OMADS7	*Oncidium* Gower Ramsey	AGL6-like	E	HM140845
*DcOSEP1*	*Dendrobium crumenatum*	SEP1/2/4-like	E	DQ119842
*DOMADS1*	*Dendrobium* Madame Thong-In	SEP1/2/4-like	E	AF198174
OMADS11	*Oncidium* Gower Ramsey	SEP1/2/4-like	E	HM140847
*DOMADS3*	*Dendrobium* Madame Thong-In	SEP3-like	E	AF198176
OMADS6	*Oncidium* Gower Ramsey	SEP3-like	E	HM140844

## References

[1] Cozzolino S, Widmer A (2005). Orchid diversity: an evolutionary consequence of deception?. Trends Ecol. Evol.

[2] Tremblay RL, Ackerman JD, Zimmerman JK, Calvo RN (2005). Variation in sexual reproduction in orchids and its evolutionary consequences: a spasmodic journey to diversification. Biol. J. Linn. Soc.

[3] Yu H, Goh CJ (2001). Molecular genetics of reproductive biology in orchids. Plant Physiol.

[4] Tsai WC, Chen HH (2006). The orchid MADS-box genes controlling floral morphogenesis. Scientific World Journal.

[5] Rudall PJ, Bateman RM (2002). Roles of synorganisation, zygomorphy and heterotopy in floral evolution: the gynostemium and labellum of orchids and other lilioid monocots. Biol. Rev. Camb. Philos. Soc.

[6] Mondragon-Palomino M, Theissen G (2009). Why are orchid flowers so diverse? Reduction of evolutionary constraints by paralogues of class B floral homeotic genes. Ann. Bot. (Lond).

[7] Cameron KM (2007). Molecular phylogenetics of Orchidaceae: the first decade of DNA sequencing.

[8] Cameron KM, Chase MW, Whitten WM, Kores PJ, Jarrell DC, Albert VA, Yukawa T, Hills HG, Goldman DH (1999). A phylogenetic analysis of the Orchidaceae: evidence from rbcL nucleotide sequences. Am. J. Bot.

[9] Gorniak M, Paun O, Chase MW (2010). Phylogenetic relationships within Orchidaceae based on a low-copy nuclear coding gene, Xdh: Congruence with organellar and nuclear ribosomal DNA results. Mol. Phylogenet. Evol.

[10] Wikstrom N, Savolainen V, Chase MW (2001). Evolution of the angiosperms: calibrating the family tree. Proc. Roy. Soc. B-Biol. Sci.

[11] Bremer K (2000). Early Cretaceous lineages of monocot flowering plants. Proc. Natl. Acad. Sci. USA.

[12] Ramirez SR, Gravendeel B, Singer RB, Marshall CR, Pierce NE (2007). Dating the origin of the Orchidaceae from a fossil orchid with its pollinator. Nature.

[13] Janssen T, Bremer K (2004). The age of major monocot groups inferred from 800+rbcL sequences. Bot. J. Linn. Soc.

[14] Gustafsson AL, Verola CF, Antonelli A (2010). Reassessing the temporal evolution of orchids with new fossils and a Bayesian relaxed clock, with implications for the diversification of the rare South American genus Hoffmannseggella (Orchidaceae: Epidendroideae). BMC Evol. Biol.

[15] Conran JG, Bannister JM, Lee DE (2009). Earliest Orchid Macrofossils: Early Miocene Dendrobium and Earina (Orchidaceae: Epidendroideae) from New Zealand. Am. J. Bot.

[16] Peakall R (2007). Speciation in the Orchidaceae: confronting the challenges. Mol. Ecol.

[17] Fu CH, Chen YW, Hsiao YY, Pan ZJ, Liu ZJ, Huang YM, Tsai WC, Chen HH (2011). OrchidBase: A collection of sequences of the transcriptome derived from orchids. Plant Cell Physiol.

[18] Schwarz-Sommer Z, Huijser P, Nacken W, Saedler H, Sommer H (1990). Genetic Control of Flower Development by Homeotic Genes in Antirrhinum majus. Science.

[19] Gramzow L, Ritz MS, Theissen G (2010). On the origin of MADS-domain transcription factors. Trends Genet.

[20] Alvarez-Buylla ER, Pelaz S, Liljegren SJ, Gold SE, Burgeff C, Ditta GS, Ribas de Pouplana L, Martinez-Castilla L, Yanofsky MF (2000). An ancestral MADS-box gene duplication occurred before the divergence of plants and animals. Proc. Natl. Acad. Sci. U S A.

[21] Parenicova L, de Folter S, Kieffer M, Horner DS, Favalli C, Busscher J, Cook HE, Ingram RM, Kater MM, Davies B, Angenent GC, Colombo L (2003). Molecular and phylogenetic analyses of the complete MADS-box transcription factor family in Arabidopsis: new openings to the MADS world. Plant Cell.

[22] Masiero S, Colombo L, Grini PE, Schnittger A, Kater MM (2011). The emerging importance of type I MADS box transcription factors for plant reproduction. Plant Cell.

[23] Riechmann JL, Meyerowitz EM (1997). MADS domain proteins in plant development. Biol. Chem.

[24] Kaufmann K, Melzer R, Theissen G (2005). MIKC-type MADS-domain proteins: structural modularity, protein interactions and network evolution in land plants. Gene.

[25] Henschel K, Kofuji R, Hasebe M, Saedler H, Munster T, Theissen G (2002). Two ancient classes of MIKC-type MADS-box genes are present in the moss Physcomitrella patens. Mol. Biol. Evol.

[26] Zobell O, Faigl W, Saedler H, Munster T (2010). MIKC* MADS-box proteins: conserved regulators of the gametophytic generation of land plants. Mol. Biol. Evol.

[27] Rounsley SD, Ditta GS, Yanofsky MF (1995). Diverse roles for MADS box genes in Arabidopsis development. Plant Cell.

[28] Alvarez-Buylla ER, Liljegren SJ, Pelaz S, Gold SE, Burgeff C, Ditta GS, Vergara-Silva F, Yanofsky MF (2000). MADS-box gene evolution beyond flowers: expression in pollen, endosperm, guard cells, roots and trichomes. Plant J.

[29] Theissen G (2001). Development of floral organ identity: stories from the MADS house. Curr. Opin. Plant Biol.

[30] Becker A, Theissen G (2003). The major clades of MADS-box genes and their role in the development and evolution of flowering plants. Mol. Phylogenet. Evol.

[31] Theissen G, Melzer R (2007). Molecular mechanisms underlying origin and diversification of the angiosperm flower. Ann. Bot.

[32] Soltis DE, Soltis PS, Albert VA, Oppenheimer DG, dePamphilis CW, Ma H, Frohlich MW, Theissen G (2002). Missing links: the genetic architecture of flowers [correction of flower] and floral diversification. Trends Plant Sci.

[33] Zhao L, Kim Y, Dinh TT, Chen X (2007). miR172 regulates stem cell fate and defines the inner boundary of APETALA3 and PISTILLATA expression domain in Arabidopsis floral meristems. Plant J.

[34] Shitsukawa N, Tahira C, Kassai K, Hirabayashi C, Shimizu T, Takumi S, Mochida K, Kawaura K, Ogihara Y, Murai K (2007). Genetic and epigenetic alteration among three homoeologous genes of a class E MADS box gene in hexaploid wheat. Plant Cell.

[35] Gramzow L, Theissen G (2010). A hitchhiker's guide to the MADS world of plants. Genome Biol.

[36] Coen ES, Meyerowitz EM (1991). The war of the whorls - genetic interactions controlling flower development. Nature.

[37] Krizek BA, Fletcher JC (2005). Molecular mechanisms of flower development: An armchair guide. Nat. Rev. Genet.

[38] Theissen G, Saedler H (2001). Plant biology. Floral quartets. Nature.

[39] Liu ZC, Mara C (2010). Regulatory mechanisms for floral homeotic gene expression. Semin. Cell Dev. Biol.

[40] Ambrose BA, Lerner DR, Ciceri P, Padilla CM, Yanofsky MF, Schmidt RJ (2000). Molecular and genetic analyses of the silky1 gene reveal conservation in floral organ specification between eudicots and monocots. Mol. Cell.

[41] Whipple CJ, Ciceri P, Padilla CM, Ambrose BA, Bandong SL, Schmidt RJ (2004). Conservation of B-class floral homeotic gene function between maize and Arabidopsis. Development.

[42] Whipple CJ, Zanis MJ, Kellogg EA, Schmidt RJ (2007). Conservation of B class gene expression in the second whorl of a basal grass and outgroups links the origin of lodicules and petals. Proc. Natl. Acad. Sci. U S A.

[43] Kim S, Koh J, Yoo MJ, Kong H, Hu Y, Ma H, Soltis PS, Soltis DE (2005). Expression of floral MADS-box genes in basal angiosperms: implications for the evolution of floral regulators. Plant J.

[44] Ferrario S, Immink RG, Angenent GC (2004). Conservation and diversity in flower land. Curr. Opin. Plant Biol.

[45] Irish VF, Litt A (2005). Flower development and evolution: gene duplication, diversification and redeployment. Curr. Opin. Genet. Dev.

[46] Malcomber ST, Kellogg EA (2005). SEPALLATA gene diversification: brave new whorls. Trends Plant Sci.

[47] Zahn LM, Kong H, Leebens-Mack JH, Kim S, Soltis PS, Landherr LL, Soltis DE, Depamphilis CW, Ma H (2005). The evolution of the SEPALLATA subfamily of MADS-box genes: a preangiosperm origin with multiple duplications throughout angiosperm history. Genetics.

[48] Litt A, Irish VF (2003). Duplication and diversification in the APETALA1/FRUITFULL floral homeotic gene lineage: implications for the evolution of floral development. Genetics.

[49] Vandenbussche M, Theissen G, Van de Peer Y, Gerats T (2003). Structural diversification and neo-functionalization during floral MADS-box gene evolution by C-terminal frameshift mutations. Nucleic Acids Res.

[50] Kyozuka J, Harcourt R, Peacock WJ, Dennis ES (1997). Eucalyptus has functional equivalents of the Arabidopsis AP1 gene. Plant Mol. Biol.

[51] Skipper M, Pedersen KB, Johansen LB, Frederiksen S, Irish VF, Johansen BB (2005). Identification and quantification of expression levels of three FRUITFULL-like MADS-box genes from the orchid Dendrobium thyrsiflorum (Reichb. f.). Plant Sci.

[52] Theissen G, Becker A, Di Rosa A, Kanno A, Kim JT, Munster T, Winter KU, Saedler H (2000). A short history of MADS-box genes in plants. Plant Mol. Biol.

[53] Ma H, Yanofsky MF, Meyerowitz EM (1991). AGL1-AGL6, an Arabidopsis gene family with similarity to floral homeotic and transcription factor genes. Genes Dev.

[54] Mena M, Mandel MA, Lerner DR, Yanofsky MF, Schmidt RJ (1995). A characterization of the MADS-box gene family in maize. Plant J.

[55] Tandre K, Albert VA, Sundas A, Engstrom P (1995). Conifer homologues to genes that control floral development in angiosperms. Plant Mol. Biol.

[56] Shindo S, Ito M, Ueda K, Kato M, Hasebe M (1999). Characterization of MADS genes in the gymnosperm Gnetum parvifolium and its implication on the evolution of reproductive organs in seed plants. Evol. Dev.

[57] Purugganan MD, Rounsley SD, Schmidt RJ, Yanofsky MF (1995). Molecular evolution of flower development: diversification of the plant MADS-box regulatory gene family. Genetics.

[58] Yu H, Goh CJ (2000). Identification and characterization of three orchid MADS-box genes of the AP1/AGL9 subfamily during floral transition. Plant Physiol.

[59] Kaufmann K, Muino JM, Jauregui R, Airoldi CA, Smaczniak C, Krajewski P, Angenent GC (2009). Target genes of the MADS transcription factor SEPALLATA3: integration of developmental and hormonal pathways in the Arabidopsis flower. PLoS Biol.

[60] Deng W, Ying H, Helliwell CA, Taylor JM, Peacock WJ, Dennis ES (2011). FLOWERING LOCUS C (FLC) regulates development pathways throughout the life cycle of Arabidopsis. Proc. Natl. Acad. Sci. U S A.

[61] Mandel MA, Yanofsky MF (1995). The Arabidopsis AGL8 MADS box gene is expressed in inflorescence meristems and is negatively regulated by APETALA1. Plant Cell.

[62] Menzel G, Apel K, Melzer S (1996). Identification of two MADS box genes that are expressed in the apical meristem of the long-day plant Sinapis alba in transition to flowering. Plant J.

[63] Bonhomme F, Sommer H, Bernier G, Jacqmard A (1997). Characterization of SaMADS D from Sinapis alba suggests a dual function of the gene: in inflorescence development and floral organogenesis. Plant Mol. Biol.

[64] Sung SK, Yu GH, An G (1999). Characterization of MdMADS2, a member of the SQUAMOSA subfamily of genes, in apple. Plant Physiol.

[65] Xu Y, Teo LL, Zhou J, Kumar PP, Yu H (2006). Floral organ identity genes in the orchid Dendrobium crumenatum. Plant J.

[66] Mandel MA, Gustafson-Brown C, Savidge B, Yanofsky MF (1992). Molecular characterization of the Arabidopsis floral homeotic gene APETALA1. Nature.

[67] Kempin SA, Savidge B, Yanofsky MF (1995). Molecular basis of the cauliflower phenotype in Arabidopsis. Science.

[68] Flanagan CA, Ma H (1994). Spatially and temporally regulated expression of the MADS-box gene AGL2 in wild-type and mutant arabidopsis flowers. Plant Mol. Biol.

[69] Savidge B, Rounsley SD, Yanofsky MF (1995). Temporal relationship between the transcription of two Arabidopsis MADS box genes and the floral organ identity genes. Plant Cell.

[70] Mandel MA, Yanofsky MF (1998). The Arabidopsis AGL9 MADS box gene is expressed in young flower primordia. Sex. Plant Reprod.

[71] Yu H, Yang SH, Goh CJ (2002). Spatial and temporal expression of the orchid floral homeotic gene DOMADS1 is mediated by its upstream regulatory regions. Plant Mol. Biol.

[72] Schwarz-Sommer Z, Hue I, Huijser P, Flor PJ, Hansen R, Tetens F, Lonnig WE, Saedler H, Sommer H (1992). Characterization of the Antirrhinum floral homeotic MADS-box gene deficiens: evidence for DNA binding and autoregulation of its persistent expression throughout flower development. Embo J.

[73] Riechmann JL, Krizek BA, Meyerowitz EM (1996). Dimerization specificity of Arabidopsis MADS domain homeotic proteins APETALA1, APETALA3, PISTILLATA, and AGAMOUS. Proc. Natl. Acad. Sci. U S A.

[74] Hill TA, Day CD, Zondlo SC, Thackeray AG, Irish VF (1998). Discrete spatial and temporal cis-acting elements regulate transcription of the Arabidopsis floral homeotic gene APETALA3. Development.

[75] Tilly JJ, Allen DW, Jack T (1998). The CArG boxes in the promoter of the Arabidopsis floral organ identity gene APETALA3 mediate diverse regulatory effects. Development.

[76] Yu H, Yang SH, Goh CJ (2000). DOH1, a class 1 knox gene, is required for maintenance of the basic plant architecture and floral transition in orchid. Plant Cell.

[77] Kramer EM, Dorit RL, Irish VF (1998). Molecular evolution of genes controlling petal and stamen development: duplication and divergence within the APETALA3 and PISTILLATA MADS-box gene lineages. Genetics.

[78] Kramer EM, Jaramillo MA, Di Stilio VS (2004). Patterns of gene duplication and functional evolution during the diversification of the AGAMOUS subfamily of MADS box genes in angiosperms. Genetics.

[79] Kramer EM, Su HJ, Wu CC, Hu JM (2006). A simplified explanation for the frameshift mutation that created a novel C-terminal motif in the APETALA3 gene lineage. BMC Evol. Biol.

[80] Chen D, Guo B, Hexige S, Zhang T, Shen D, Ming F (2007). SQUA-like genes in the orchid Phalaenopsis are expressed in both vegetative and reproductive tissues. Planta.

[81] Ferrandiz C, Gu Q, Martienssen R, Yanofsky MF (2000). Redundant regulation of meristem identity and plant architecture by FRUITFULL, APETALA1 and CAULIFLOWER. Development.

[82] Fornara F, Parenicova L, Falasca G, Pelucchi N, Masiero S, Ciannamea S, Lopez-Dee Z, Altamura MM, Colombo L, Kater MM (2004). Functional characterization of OsMADS18, a member of the AP1/SQUA subfamily of MADS box genes. Plant Physiol.

[83] Gocal GF, King RW, Blundell CA, Schwartz OM, Andersen CH, Weigel D (2001). Evolution of floral meristem identity genes. Analysis of Lolium temulentum genes related to APETALA1 and LEAFY of Arabidopsis. Plant Physiol.

[84] Gu Q, Ferrandiz C, Yanofsky MF, Martienssen R (1998). The FRUITFULL MADS-box gene mediates cell differentiation during Arabidopsis fruit development. Development.

[85] Immink RG, Hannapel DJ, Ferrario S, Busscher M, Franken J, Lookeren Campagne MM, Angenent GC (1999). A petunia MADS box gene involved in the transition from vegetative to reproductive development. Development.

[86] Huijser P, Klein J, Lonnig WE, Meijer H, Saedler H, Sommer H (1992). Bracteomania, an inflorescence anomaly, is caused by the loss of function of the MADS-box gene squamosa in Antirrhinum majus. Embo J.

[87] Irish VF, Sussex IM (1990). Function of the apetala-1 gene during Arabidopsis floral development. Plant Cell.

[88] Gustafson-Brown C, Savidge B, Yanofsky MF (1994). Regulation of the arabidopsis floral homeotic gene APETALA1. Cell.

[89] Berbel A, Navarro C, Ferrandiz C, Canas LA, Madueno F, Beltran JP (2001). Analysis of PEAM4, the pea AP1 functional homologue, supports a model for AP1-like genes controlling both floral meristem and floral organ identity in different plant species. Plant J.

[90] Hsu HF, Huang CH, Chou LT, Yang CH (2003). Ectopic expression of an orchid (Oncidium Gower Ramsey) AGL6-like gene promotes flowering by activating flowering time genes in Arabidopsis thaliana. Plant Cell Physiol.

[91] Mandel MA, Yanofsky MF (1995). A gene triggering flower formation in Arabidopsis. Nature.

[92] Pelaz S, Gustafson-Brown C, Kohalmi SE, Crosby WL, Yanofsky MF (2001). APETALA1 and SEPALLATA3 interact to promote flower development. Plant J.

[93] Honma T, Goto K (2001). Complexes of MADS-box proteins are sufficient to convert leaves into floral organs. Nature.

[94] Hsu HF, Yang CH (2002). An orchid (Oncidium Gower Ramsey) AP3-like MADS gene regulates floral formation and initiation. Plant Cell Physiol.

[95] Hsu HF, Hsieh WP, Chen MK, Chang YY, Yang CH (2010). C/D Class MADS Box Genes from Two Monocots, Orchid (Oncidium Gower Ramsey) and Lily (Lilium longiflorum), Exhibit Different Effects on Floral Transition and Formation in Arabidopsis thaliana. Plant Cell Physiol.

[96] Chang YY, Chiu YF, Wu JW, Yang CH (2009). Four orchid (Oncidium Gower Ramsey) AP1/AGL9-like MADS box genes show novel expression patterns and cause different effects on floral transition and formation in Arabidopsis thaliana. Plant Cell Physiol.

[97] Angenent GC, Busscher M, Franken J, Mol JN, van Tunen AJ (1992). Differential expression of two MADS box genes in wild-type and mutant petunia flowers. Plant Cell.

[98] Angenent GC, Franken J, Busscher M, Weiss D, van Tunen AJ (1994). Co-suppression of the petunia homeotic gene fbp2 affects the identity of the generative meristem. Plant J.

[99] Pnueli L, Hareven D, Broday L, Hurwitz C, Lifschitz E (1994). The TM5 MADS Box Gene Mediates Organ Differentiation in the Three Inner Whorls of Tomato Flowers. Plant Cell.

[100] Pelaz S, Ditta GS, Baumann E, Wisman E, Yanofsky MF (2000). B and C floral organ identity functions require SEPALLATA MADS-box genes. Nature.

[101] Tzeng TY, Hsiao CC, Chi PJ, Yang CH (2003). Two lily SEPALLATA-like genes cause different effects on floral formation and floral transition in Arabidopsis. Plant Physiol.

[102] Moon YH, Kang HG, Jung JY, Jeon JS, Sung SK, An G (1999). Determination of the motif responsible for interaction between the rice APETALA1/AGAMOUS-LIKE9 family proteins using a yeast two-hybrid system. Plant Physiol.

[103] Kyozuka J, Kobayashi T, Morita M, Shimamoto K (2000). Spatially and temporally regulated expression of rice MADS box genes with similarity to Arabidopsis class A, B and C genes. Plant Cell Physiol.

[104] Masiero S, Imbriano C, Ravasio F, Favaro R, Pelucchi N, Gorla MS, Mantovani R, Colombo L, Kater MM (2002). Ternary complex formation between MADS-box transcription factors and the histone fold protein NF-YB. J. Biol. Chem.

[105] Chen MK, Lin IC, Yang CH (2008). Functional analysis of three lily (Lilium longiflorum) APETALA1-like MADS box genes in regulating floral transition and formation. Plant Cell Physiol.

[106] Zhang P, Tan HT, Pwee KH, Kumar PP (2004). Conservation of class C function of floral organ development during 300 million years of evolution from gymnosperms to angiosperms. Plant J.

[107] Skipper M, Johansen LB, Pedersen KB, Frederiksen S, Johansen BB (2006). Cloning and transcription analysis of an AGAMOUS- and SEEDSTICK ortholog in the orchid Dendrobium thyrsiflorum (Reichb. f.). Gene.

[108] Lopez-Dee ZP, Wittich P, Enrico Pe M, Rigola D, Del Buono I, Gorla MS, Kater MM, Colombo L (1999). OsMADS13, a novel rice MADS-box gene expressed during ovule development. Dev. Genet.

[109] Xu HY, Li XG, Li QZ, Bai SN, Lu WL, Zhang XS (2004). Characterization of HoMADS 1 and its induction by plant hormones during *in vitro* ovule development in Hyacinthus orientalis L. Plant Mol. Biol.

[110] Tzeng TY, Chen HY, Yang CH (2002). Ectopic expression of carpel-specific MADS box genes from lily and lisianthus causes similar homeotic conversion of sepal and petal in Arabidopsis. Plant Physiol.

[111] Schmidt RJ, Veit B, Mandel MA, Mena M, Hake S, Yanofsky MF (1993). Identification and molecular characterization of ZAG1, the maize homolog of the Arabidopsis floral homeotic gene AGAMOUS. Plant Cell.

[112] Pinyopich A, Ditta GS, Savidge B, Liljegren SJ, Baumann E, Wisman E, Yanofsky MF (2003). Assessing the redundancy of MADS-box genes during carpel and ovule development. Nature.

[113] Rigola D, Pe ME, Fabrizio C, Me G, Sari-Gorla M (1998). CaMADS1, a MADS box gene expressed in the carpel of hazelnut. Plant Mol. Biol.

[114] Song IJ, Nakamura T, Fukuda T, Yokoyama J, Ito T, Ichikawa H, Horikawa Y, Kameya T, Kanno A (2006). Spatiotemporal expression of duplicate AGAMOUS orthologues during floral development in Phalaenopsis. Dev. Genes Evol.

[115] Kang HG, Noh YS, Chung YY, Costa MA, An K, An G (1995). Phenotypic alterations of petal and sepal by ectopic expression of a rice MADS box gene in tobacco. Plant Mol. Biol.

[116] Rutledge R, Regan S, Nicolas O, Fobert P, Cote C, Bosnich W, Kauffeldt C, Sunohara G, Seguin A, Stewart D (1998). Characterization of an AGAMOUS homologue from the conifer black spruce (Picea mariana) that produces floral homeotic conversions when expressed in Arabidopsis. Plant J.

[117] Angenent GC, Franken J, Busscher M, van Dijken A, van Went JL, Dons HJ, van Tunen AJ (1995). A novel class of MADS box genes is involved in ovule development in petunia. Plant Cell.

[118] Colombo L, Franken J, Koetje E, van Went J, Dons HJ, Angenent GC, van Tunen AJ (1995). The petunia MADS box gene FBP11 determines ovule identity. Plant Cell.

[119] Zahn LM, Leebens-Mack J, DePamphilis CW, Ma H, Theissen G (2005). To B or Not to B a flower: the role of DEFICIENS and GLOBOSA orthologs in the evolution of the angiosperms. J. Hered.

[120] Tsai WC, Kuoh CS, Chuang MH, Chen WH, Chen HH (2004). Four DEF-like MADS box genes displayed distinct floral morphogenetic roles in Phalaenopsis orchid. Plant Cell Physiol.

[121] Tsai WC, Lee PF, Chen HI, Hsiao YY, Wei WJ, Pan ZJ, Chuang MH, Kuoh CS, Chen WH, Chen HH (2005). PeMADS6, a GLOBOSA/PISTILLATA-like gene in Phalaenopsis equestris involved in petaloid formation, and correlated with flower longevity and ovary development. Plant Cell Physiol.

[122] Tsai WC, Pan ZJ, Hsiao YY, Jeng MF, Wu TF, Chen WH, Chen HH (2008). Interactions of B-class complex proteins involved in tepal development in Phalaenopsis orchid. Plant Cell Physiol.

[123] Kim S-Y, Yun P-Y, Fukuda T, Ochiai T, Yokoyama J, Kameya T, Kanno A (2007). Expression of a DEFICIENS-like gene correlates with the differentiation between sepal and petal in the orchid, Habenaria radiata (Orchidaceae). Plant Sci.

[124] Chang YY, Kao NH, Li JY, Hsu WH, Liang YL, Wu JW, Yang CH (2010). Characterization of the possible roles for B class MADS box genes in regulation of perianth formation in orchid. Plant Physiol.

[125] Yu D, Kotilainen M, Pollanen E, Mehto M, Elomaa P, Helariutta Y, Albert VA, Teeri TH (1999). Organ identity genes and modified patterns of flower development in Gerbera hybrida (Asteraceae). Plant J.

[126] Aceto S, Montieri S, Sica M, Gaudio L (2007). Molecular evolution of the OrcPI locus in natural populations of Mediterranean orchids. Gene.

[127] Cantone C, Sica M, Gaudio L, Aceto S (2009). The OrcPI locus: Genomic organization, expression pattern, and noncoding regions variability in Orchis italica (Orchidaceae) and related species. Gene.

[128] Aceto S, Cantone C, Chiaiese P, Ruotolo G, Sica M, Gaudio L (2010). Isolation and phylogenetic footprinting analysis of the 5'-regulatory region of the floral homeotic gene OrcPI from Orchis italica (Orchidaceae). J. Hered.

[129] Cantone C, Gaudio L, Aceto S (2011). The GLO-like locus in orchids: duplication and purifying selection at synonymous sites within Orchidinae (Orchidaceae). Gene.

[130] Mondragon-Palomino M, Hiese L, Harter A, Koch MA, Theissen G (2009). Positive selection and ancient duplications in the evolution of class B floral homeotic genes of orchids and grasses. BMC Evol. Biol.

[131] Kanno A, Saeki H, Kameya T, Saedler H, Theissen G (2003). Heterotopic expression of class B floral homeotic genes supports a modified ABC model for tulip (Tulipa gesneriana). Plant Mol. Biol.

[132] Nakamura T, Fukuda T, Nakano M, Hasebe M, Kameya T, Kanno A (2005). The modified ABC model explains the development of the petaloid perianth of Agapanthus praecox ssp. orientalis (Agapanthaceae) flowers. Plant Mol. Biol.

[133] Mondragon-Palomino M, Theissen G (2008). MADS about the evolution of orchid flowers. Trends Plant Sci.

[134] Mondragon-Palomino M, Theissen G (2011). Conserved differential expression of paralogous DEFICIENS- and GLOBOSA-like MADS-box genes in the flowers of Orchidaceae: refining the 'orchid code'. Plant J.

